# Age‐dependent alterations in osteoblast and osteoclast activity in human cancellous bone

**DOI:** 10.1111/jcmm.13192

**Published:** 2017-04-26

**Authors:** Mustafa Becerikli, Henriette Jaurich, Jessica Schira, Matthias Schulte, Carmen Döbele, Christoph Wallner, Stephanie Abraham, Johannes M. Wagner, Mehran Dadras, Ulrich Kneser, Marcus Lehnhardt, Björn Behr

**Affiliations:** ^1^ Department of Plastic Surgery BG University Hospital Bergmannsheil Ruhr‐University Bochum Bochum Germany; ^2^ Department of Plastic Surgery BG Trauma Hospital Ludwigshafen University of Heidelberg Ludwigshafen Germany

**Keywords:** bone ageing, osteoblasts, osteoclasts, *RUNX‐2*, *RANKL*

## Abstract

It is assumed that the activity of osteoblasts and osteoclasts is decreased in bone tissue of aged individuals. However, detailed investigation of the molecular signature of human bone from young compared to aged individuals confirming this assumption is lacking. In this study, quantitative expression analysis of genes related to osteogenesis and osteoclastogenesis of human cancellous bone derived from the distal radius of young and aged individuals was performed. Furthermore, we additionally performed immunohistochemical stainings. The young group included 24 individuals with an average age of 23.2 years, which was compared to cancellous bone derived from 11 body donators with an average age of 81.0 years. In cancellous bone of young individuals, the osteogenesis‐related genes *RUNX‐2*,*OSTERIX*,*OSTEOPONTIN* and *OSTEOCALCIN* were significantly up‐regulated compared to aged individuals. In addition, *RANKL* and *NFATc1*, both markers for osteoclastogenesis, were significantly induced in cancellous bone of young individuals, as well as the WNT gene family member *WNT5a* and the matrix metalloproteinases *MMP‐9*. However, quantitative RT‐PCR analysis of *BMP‐2*,*ALP*,*FGF‐2*,*CYCLIN‐D1*,*MMP‐13*,*RANK*,*OSTEOPROTEGERIN* and *TGFb1* revealed no significant difference. Furthermore, Tartrate‐resistant acid phosphatase (TRAP) staining was performed which indicated an increased osteoclast activity in cancellous bone of young individuals. In addition, pentachrome stainings revealed significantly less mineralized bone matrix, more osteoid and an increased bone density in young individuals. In summary, markers related to osteogenesis as well as osteoclastogenesis were significantly decreased in the aged individuals. Thus, the present data extends the knowledge about reduced bone regeneration and healing capacity observed in aged individuals.

## Introduction

Bone remodelling is a dynamic process which starts at the early embryonic stage and sustains throughout life‐time and is tightly regulated by bone‐building osteoblasts (OB) and bone resorbing osteoclasts (OC). During ageing, bone homoeostasis seems to be affected by progressive deprivation of cell function and proliferation 1, 2. During ageing, regulation of bone remodelling could be impaired resulting in decreased bone density and fragile bone structure. Previous studies indicated that aged individuals have a 10‐fold increased fracture risk compared to young individuals 3, 4. Demographic data indicated that 79% of the total German population is younger than 65 years (Web Reference 1). Of those, the German Federal Health Reporting in 2014 stated 360,000 bone fractures (Web Reference 2). In contrast, 21% representing individuals older than 65 years included 470,000 fractures, which clearly indicate the overrepresentation of fractures in the respective subpopulation. Changing of composition of the society′s age indicates that the risk of fractures leading to impaired bone healing steadily increases, which makes a better understanding of bone ageing and homoeostasis indispensable.

Accordingly, studies in rodents showed that bone homoeostasis and healing is age dependent. Analysis of bone regeneration in a calvarial defect mice model revealed that young mice have a better bone healing capacity than aged mice 5, 6, which could depend on increased sclerostin expression in adult mice. In addition, Kavukcuoglu and coworkers showed a diminished bone density and elasticity in aged mice 7. Furthermore, analyses of young and aged rats revealed a significant age‐dependent decrease in bone mineral density (BMD) and less proliferating OB 8.

*In vitro* studies further confirmed age‐related differences in differentiation of OB. Differentiation of bone marrow cells isolated from 6 weeks, 6 months and 18 months old mice revealed that OB differentiation is significantly reduced in aged mice compared to young and middle aged mice 9. In addition, mature osteoblasts of aged compared with those of young mice showed enhanced expression of Wnt9b and decreased expression of Wnt5a and 7b. In early osteoblasts, mRNA levels of Wnt1, 5a, 5b, and 7b were significantly increased in aged mice 9. Similarly, cells isolated from bone marrow of rhesus monkeys show a decreased osteogenic differentiation potential isolated from the 8–12 year old animals compared to cells from animals younger than 5 years 10. This was also shown in a human study, comparing osteogenic differentiation potential of bone marrow‐derived stem cells (BMSCs) of young and aged patients 11. Age also has an effect on osteogenic differentiation potential of human adipose‐derived stem cells (hASCs). ASCs isolated from adipose tissue obtained from patients in the age ranging between 20 and 58 years showed an age‐dependent decrease in osteogenic differentiation potential 12.

Interestingly, only few studies decipher the molecular differences between young and old in human bone tissue. Comparison of mesenchymal stem cells (hMSCs) obtained from groups of young (<50 years) and aged (>55 years) individuals indicated an increased *WNT* gene expression in the young group compared to the aged group. Particularly, *WNT5A* expression in hMSCs from young women was significantly increased compared to aged women 13.

Thus, molecular changes in bone homoeostasis during ageing are poorly defined. In the present study, we performed a detailed analyses of numerous osteogenic and osteoclastogenic related markers in human cancellous bone derived from young and aged individuals to characterize age‐dependent differences in bone remodelling and homoeostasis.

## Materials and methods

### Human specimens and tissue processing

Tissue harvest and experiments were performed in accordance with the ethical committees, and informed consent was obtained from the patients. For this study, 24 patients with scaphoid fracture and subsequent surgical intervention including bone transplantation from the ipsilateral radius were recruited from two regional hand trauma centres (young group). Additionally, cancellous bone of eleven body donors was harvested (aged group). The average age of the young group was 23.2 (range between 18 and 35 years). In contrast, the average age of the aged group was 81.0 (range between 62 and 95 years). Of note, no anti‐resorptive agent or corticosteroid intake was filed in the medical records of the body donors. Table S1 summarizes the medical conditions of the body donors. Tissue of body donors was harvested immediately, before any fixating steps after death. After removal, tissue was immediately washed in ice‐cold PBS to avoid contaminations from blood cells. Afterwards, tissue was either frozen at −80°C until RNA preparation or directly processed for histology.

### RNA preparation and cDNA synthesis

RNA preparation and cDNA synthesis was performed as previously described 14. Briefly, tissue was homogenized with Polytron homogenizer (Kinematica, Eschbach, Germany) in 1 ml TRIzol reagent (Thermo Fisher Scientific, Darmstadt, Germany) on ice. After incubation at room temperature for 5 min., 200 μl chloroform (Merck, Darmstadt, Germany) was added and mixed for 5 sec. Subsequently, samples were centrifuged at 15,300 r.p.m. for 15 min. at 4°C and the aqueous phase was processed for RNA isolation. After addition of 1 μl glycogen (Roche, Mannheim, Germany) as a carrier and 250 μl of 100% isopropanol, samples were incubated at −80°C over night. After centrifugation at 12,000 r.p.m. for 30 min. at 4°C, pellets were washed with 1 ml 75% ethanol and again centrifuged at 12,000 r.p.m. for 5 min. at 4°C. Finally, RNA was air‐dried for 20 min., resuspended in 100 μl RNase‐free water and incubated at 60°C for 10 min. To gain high purity, RNA clean up was performed with RNeasy Mini Kit (Qiagen, Hilden, Germany) according to manufacturer's instructions including DNase digestion to avoid genomic DNA contaminations (RNase free DNase Kit; Qiagen). For reverse transcription, 200 ng total RNA per reaction was transcribed using the High Capacity cDNA Reverse Transcription Kit with RNase inhibitor (Thermo Fisher Scientific) following the manufacturer's instructions.

### Quantitative real‐time PCR (qRT‐PCR)

Quantitative relative gene expression was determined on Applied Biosystems StepOnePlus real‐time PCR System using TaqMan® gene expression assays (genes and assay IDs are listed in Table 1) and TaqMan® universal master mix (Applied Biosystems, Darmstadt, Germany). For quantitative analysis, 2 μl cDNA were used for each reaction. Data were analysed according to the manufacturer's ΔΔC_t_ method (Applied Biosystems) using *18S* as reference gene. Each sample of young individuals was related to the mean expression value of cancellous bone samples from aged individuals.

**Table 1:** Genes *and* TaqMan® gene expression assay IDs

*18S*	Hs99999901_s1
*ALP*	Hs01029144_m1
*BMP2*	Hs00154192_m1
*CCND1*	Hs00765553_m1
*FGF2*	Hs00266645_m1
*MMP9*	Hs00234579_m1
*MMP13*	Hs00233992_m1
*NFATc1*	Hs00542678_m1
*Noggin*	Hs00271352_s1
*OCN*	Hs01587814_g1
*OPG*	Hs00900358_m1
*OPN*	Hs00959010_m1
*Osterix*	Hs01866874_s1
*RANK*	Hs00921372_m1
*RANKL*	Hs00243522_m1
*RUNX2*	Hs00231692_m1
*TGFb1*	Hs00998133_m1
*TNF‐a*	Hs01113624_g1
*WNT5a*	Hs00998537_m1

### Histology and immunostaining

Harvested tissue was shortly washed with cold PBS to avoid contaminations from blood cells, fixed in 4% paraformaldehyde (Sigma‐Aldrich, St. Louis, MO, USA) overnight at 4°C and decalcified in 19% EDTA (Applichem, Darmstadt, Germany) for 7 days. After dehydration and embedding in paraffin, bone tissue was cut into serial sections of 9 μm. After antigen retrieval with proteinase K, immunohistochemical staining for alkaline phosphatase (ALP; #sc166261; Santa Cruz Biotechnology, Heidelberg, Germany, dilution 1:100;), RANKL (#ab45039; abcam, Cambridge, United Kingdom, dilution 1:100), osteocalcin (#sc30045; Santa Cruz Biotechnology, dilution 1:100) and RUNX2 (#sc10758; Santa Cruz Biotechnology, dilution 1:100) was performed overnight at 4°C. Isotype controls were performed with mouse IgG1 or polyclonal rabbit isotype control antibodies (Thermo Fisher Scientific, dilution 1:100). Subsequently, sections were incubated with antimouse Alexa Fluor 488, anti‐rabbit Alexa Fluor 594 or anti‐rabbit Alexa Fluor 594 secondary antibody (Thermo Fisher Scientific) for 2 hrs at RT, respectively, performing DAPI counterstaining in parallel. For each protein, immunofluorescence was performed on samples from all eleven body donators and at least six young individuals.

Pentachrome staining was performed as previously described 15. TRAP staining was performed with a leucocyte acid phosphatase kit (#387A‐1KT; Sigma‐Aldrich). For the analysis of immunohistochemical stainings as well as for quantification of bone volume/total volume (BV/TV), an inverse Olympus X83 microscope was used and the number of pixels was quantified using the software Photoshop (Adobe Systems, San Jose, California). Results are shown in arbitrary units.

### Statistical analysis

Results of qRT‐PCR and histological experiments were given as mean ± S.E.M. Statistical analyses were performed by unpaired 2‐tailed student's *t*‐test. *P*‐values <0.05 were considered statistically significant and indicated in the figures as follows: *: *P* < 0.05; **: *P* < 0.01; ***: *P* < 0.001.

## Results

### Expression of osteogenesis‐related genes in aged and young bone

**Figure 1:**
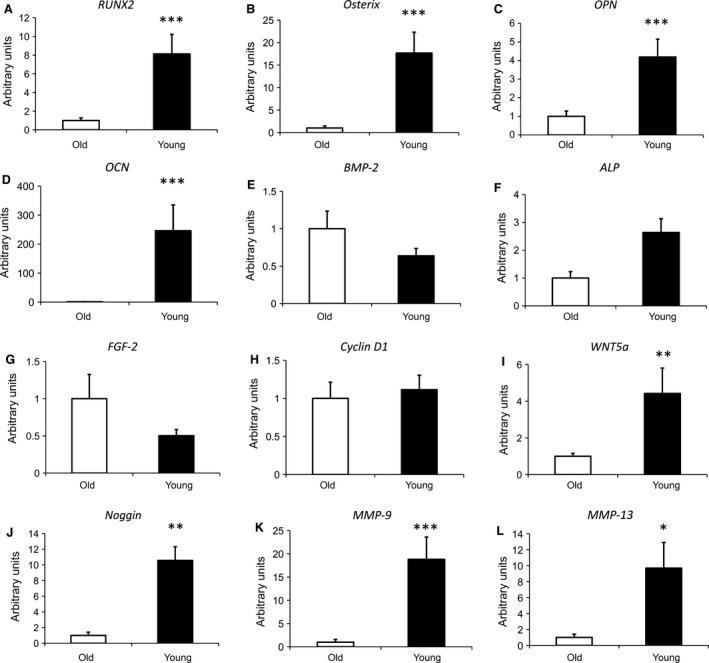
Quantitative RT‐PCR analysis of osteogenesis‐related genes in human cancellous bone of aged and young individuals. RUNX2 (**A**), *Osterix* (**B**), OPN (**C**), OCN (**D**), BMP‐2 (**E**), ALP (**F**), FGF‐2 (**G**), Cyclin D1 (**H**) WNT5a (**I**), Noggin (**J**), MMP‐9 (**K**), MMP‐13 (**L**). Samples (aged *n* = 11; young *n* = 17) were normalized to the housekeeping gene 18S rRNA and data are presented as mean ± S.E.M.; Student's *t*‐test. (*P < 0.05, **P < 0.01, ***P < 0.001).

We performed quantitative RT‐PCR analyses to obtain expression profiles of osteogenesis and osteoclastogenesis‐related genes of human cancellous bone from young and aged individuals. Runt‐related transcription factor 2 (*RUNX‐2*) is a key transcription factor regulating differentiation of OB 16, 17, 18. Expression of *RUNX‐2* was significantly up‐regulated (~ 8‐fold increase) in cancellous bone of young compared to aged individuals (Fig. 1A). *OSTERIX* which is required for OB differentiation and bone formation 19, 20 and a downstream target of *RUNX‐2* was likewise significantly increased (Fig. 1B). Additionally, expression patterns of late OB differentiation markers *OPN* and *OCN,* which play important roles in bone metabolism and remodelling 21, 22, 23 were both up‐regulated in young individuals (Fig. 1C and D). Remarkably, OCN which is secreted exclusively by OB and implicated in bone mineralization was 240‐fold increased. In contrast, the growth factors *BMP‐2* and *FGF‐2*, both essential for OB proliferation and bone formation 24, 25, showed similar expression patterns in both groups (Fig. 1E and G). Similarly, the OB differentiation marker *ALP* and *CYCLIN‐D1,* required for cell cycle progression were not differentially regulated (Fig. 1F and H). Interestingly, expression of the BMP inhibitor *Noggin* 26 as well as *WNT5A,* which is involved in regulating osteoblastic differentiation and osteoclastogenesis 27, 28 was significantly increased in bone of young individuals (Fig. 1I and J). In addition, the matrix metalloproteinases *MMP‐9* and *MMP‐13*, which play a role in angiogenesis and bone remodelling 29, were up‐regulated in cancellous bone of young individuals (Fig. 1K and L).

### Expression of osteoclastogenesis‐ and immune response‐related genes

**Figure 2:**
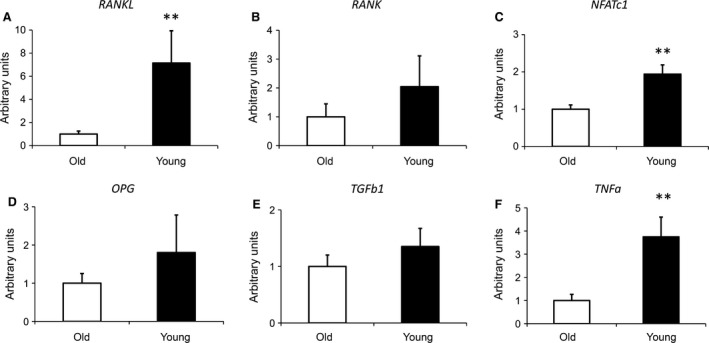
Quantitative RT‐PCR analysis of osteoclastogenesis‐related genes in human cancellous bone of old and young individuals. RANKL (**A**), RANK (**B**), NFATc1 (**C**) OPG (**D**), TGFb1 (**E**), TNFα (**F**). Samples (aged *n* = 11; young *n* = 17) were normalized to the housekeeping gene 18S rRNA and data are presented as mean ± S.E.M.; Student's *t*‐test. (**P < 0.01).

OC differentiation is promoted by binding of receptor activator of nuclear factor‐κB ligand (RANKL) 30 to its receptor RANK 31. Among others, downstream effector is the transcription factor nuclear factor of activated T cells c (NFATc1), which could induce the expression of OC‐specific genes 32. We performed quantitative RT‐PCR analyses to investigate, whether these key molecules of osteoclastogenesis are differentially regulated in cancellous bone of young persons compared to cancellous bone of the aged group. *RANKL* was seven‐fold up‐regulated in cancellous bone of young individuals (Fig. 2A). The RANKL receptor *RANK* was moderately but not significantly up‐regulated (Fig. 2B). In addition, *NFATc1* expression showed a slight increase (Fig. 2C). The secreted glycoprotein *osteoprotegerin* (*OPG*), which is a RANKL antagonist blocking OC differentiation from precursor cells 33 as well as the transforming growth factor β (*TGF*β*1*), which promotes osteoclastogenesis 34 were not differentially expressed (Fig. 2D and E). Interestingly, expression of *TNF*α, which could promote osteoclastogenesis 35 was significantly up‐regulated in cancellous bone of young individuals compared to the aged group (Fig. 2F).

### Staining of osteogenesis‐ and osteoclastogenesis‐related markers

**Figure 3:**
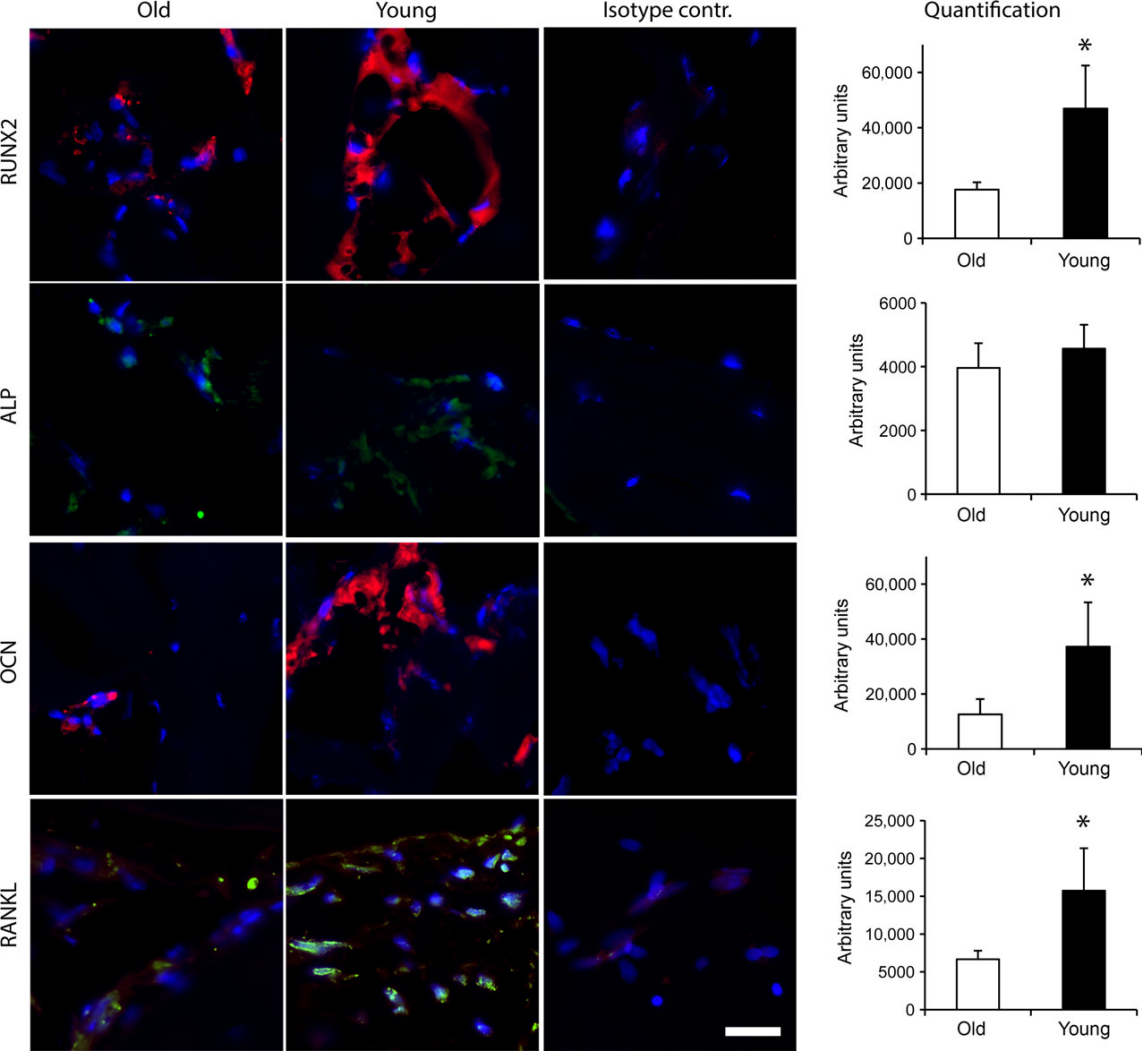
Immunohistochemical staining of osteogenesis‐ and osteoclastogenesis‐related proteins in human cancellous bone of old and young individuals. Sample size for aged individuals was *n* = 11 (RUNX2), 8 (ALP), 11 (OCN) and 11 (RANKL); for young individuals *n* = 5 (*RUNX*2), 6 (ALP), 5 (OCN) and 5 (RANKL). Additionally, the isotype control for each type of antibody is shown. Data are presented as mean ± S.E.M.; Student's *t*‐test. (*P < 0.05). Scale bar: 20 μm.

**Figure 4:**
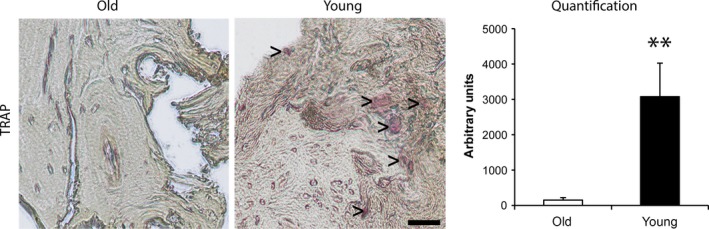
Histological staining of Tartrate‐resistant acid phosphatase (TRAP)‐positive OCs. TRAP‐positive OCs are indicated by>. Sample size was *n* = 10 for aged individuals and *n* = 6 for young individuals. Data are presented as mean ± S.E.M.; Student's *t*‐test. (** *P* < 0.01). Scale bar: 50 μm.

For validation of gene expression data, immunohistochemical stainings of osteogenesis‐ and osteoclastogenesis‐related markers were performed. RUNX‐2 protein was detected to be significantly up‐regulated in cancellous bone of the young group compared to aged individuals, which confirms quantitative gene expression data (Fig. 3). In accordance with quantitative RT‐PCR data, no difference in ALP activity was observed. Furthermore, OCN as well as RANKL protein expression was induced in young individuals compared to the aged group confirming gene expression data. Of note, no staining was observed in isotype control experiments (Fig. 3). In addition, comparison of young and aged individuals indicated a significant increase of TRAP‐positive cells in cancellous bone of young individuals indicating an increased activity of OCs which likewise confirms increased expression of osteclastogenesis‐related genes (Fig. 4).

### Altered architecture and BV/TV in old *versus* young bone

**Figure 5:**
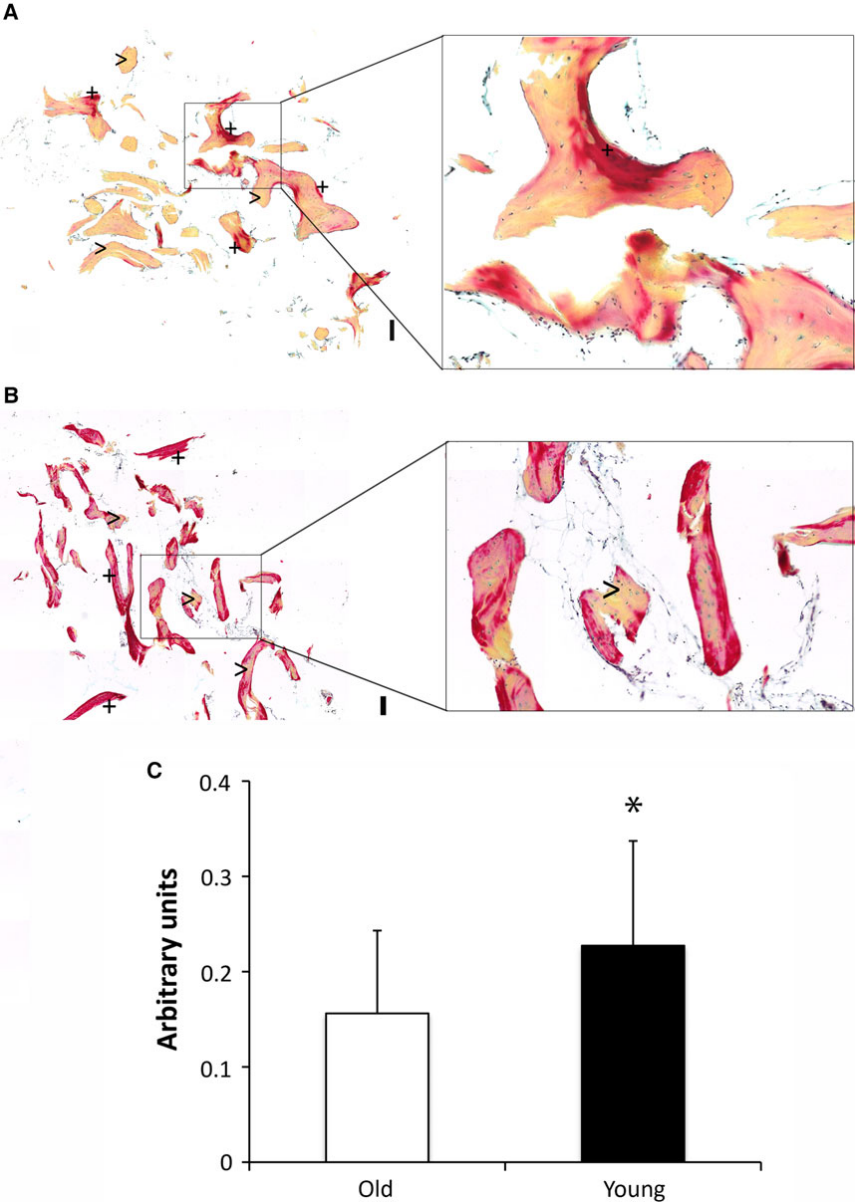
Architecture of aged (**A**) compared young bone (**B**) shown by pentachrome staining. Mineralized bone structures is visualized in yellow (indicated by>); osteoid in red (indicated by +). BV/TV analysis (**C**). Sample size was *n* = 11 for aged individuals and *n* = 24 for young individuals. Data are presented as mean ± S.E.M.; Student's *t*‐test. (*P < 0.05). Scale bar: 200 μm.

For comparison of the bone architecture of young and aged individuals, pentachrome stainings were performed. Bone of aged individuals showed a high proportion of mineralized bone matrix (Fig. 5A). In contrast, a high proportion of osteoid as well as significantly decreased proportion of mineralized bone matrix was observed in bone of young individuals (Fig. 5B). Additionally, quantitative analysis of bone volume normalized to total volume (BV/TV) was performed. Bone density in cancellous bone of young individuals was significantly up‐regulated (~ 1,5‐fold increase) compared to the aged group (Fig. 5C).

## Discussion

Tightly regulated bone remodelling could become unbalanced due to ageing which results in higher fracture risk and impaired bone healing 9, 36. As detailed information about the molecular mechanisms leading to altered bone homoeostasis in aged human bone is rare, we analysed the expression of several osteogenesis‐ and osteoclastogenesis‐related markers by quantitative RT‐PCR, histology and immunohistochemistry in human cancellous bone of young and aged donors. Several genes regulating osteogenesis were down‐regulated in cancellous bone of aged individuals. Beside *RUNX‐2,* which is a key transcription factor for osteoblastic differentiation, gene expression of the downstream targets *osterix*,* OPN* and *OCN* were likewise decreased in aged individuals indicating that osteoblast differentiation and bone formation is impaired. In contrast, *ALP* expression was not differentially regulated in aged bone compared to the young group. *BMP‐2*, which plays a role in bone repair and initiation of fracture healing, was slightly reduced in bone of young individuals which could be due to high expression levels of the BMP antagonist *Noggin WNT5A* which is involved in both osteoblastic differentiation and osteoclastogenesis was increased in the young group indicating an activation of both processes. In accordance with our results, Shen *et al*. observed that hMSCs derived from young individuals show an up‐regulation of *WNT*‐genes including *WNT5A* compared to hMSCs derived from aged bone 13. Matrix metalloproteinases are necessary for a healthy bone physiology and remodelling. The down‐regulation of *MMP‐9* and *MMP‐13* in bone of aged persons is a hint for a disturbed balance in bone remodelling and physiology.

Additionally, expression analysis of the osteoclastogenesis markers *RANKL* and *NFATc1* as well as increased TRAP staining indicate enhanced osteoclast activation in cancellous bone of young individuals compared to the aged group. Thus, in contrast to aged bone, young bone shows elevated osteoblast as well as osteoclast activity. Moreover, bone density was significantly higher in cancellous bone of young individuals compared to the aged group. Furthermore, pentachrome staining revealed that young bone contains more osteoid and non‐mineralized bone matrix whereas aged bone is mainly mineralized and thus less elastic. No difference in *CYCLIN‐D1* expression was observed which indicates that cell proliferation in bone of old and young persons is not significantly different.

About 10% of bone volume is replaced by continuous bone remodelling within 1 year 37. With increasing age, less bone is replaced during each remodelling cycle while an increased amount of non‐calcified collagenous matrix is denatured 38, 39. Bone tissue is consisting of the mineral matrix, which provides stiffness and strength and the organic matrix with collagen as the main component that allows ductility and toughness. During ageing, bone collagen and mineral concentrations show an inverse progression 40. Analyses of age‐related changes of cortical bone from 47 human body donors with an age range between 20 and 102 years revealed a markedly decrease of mechanical properties and a significant increase of porosity with age 41. Interestingly, investigation of the three‐dimensional microarchitecture of cancellous bone from adolescents (9 to 17 years), young adults (18 to 24 years) and adults (25 to 30 years) by micro‐CT analyses revealed a similar BV/TV for adolescent and adult cancellous bone 40. Furthermore, similar to our results, analyses showed significantly higher collagen concentration and reciprocal lower mineral concentration in the adolescent cancellous bone than in the adult cancellous bone 40. Additionally, similar to our RT‐PCR results, significantly reduced OCN protein expression was also observed in osteoblastic cells derived from aged compared to young individuals 42, indicating impaired bone formation.

The age of the young group investigated in the present study ranged between 18 and 35 years. In summary, we examined bone from young and aged human and demonstrated a significant decrease in both osteoblastic and osteoclastic processes in aged individuals without a conspicuous alteration in the balance. Many osteogenesis‐related genes were significantly down‐regulated compared to young individuals. Additionally, markers for osteoclastogenesis, as well as bone density were also significantly reduced in cancellous bone of aged individuals. These data contribute to our understanding for the deranged bone renewal and healing performance noticed in old persons.

## Conflict of interest

The authors declare that no conflict of interest and financial disclosure exists.

## Author's contribution

M.B. analysed quantitative RT‐PCR data and stainings including statistics, prepared figures and wrote the manuscript; H.J. performed stainings and qRT‐PCR; J.S. analysed data and wrote the manuscript; M.S. contributed to tissue analysis, to tissue collection and preparation; C.D. performed stainings; C.W. prepared figures and contributed to the manuscript; S.A. performed stainings and qRT‐PCR; J.M.W., M.D.; U.K. and M.L. provided tissue and supervised the study; B.B. designed the research study, analysed stainings and wrote the manuscript.

### Web references

http://www.bib-demografie.de/DE/ZahlenundFakten/02/Abbildungen/a_02_12_ag_20_65_80_d_1871_2060.html?nn=3074114. Reference was last accessed on May 27th 2016.

http://www.gbe-bund.de/oowa921-install/servlet/oowa/aw92/dboowasys921.xwdevkit/xwd_init?gbe.isgbetol/xs_start_neu/&p_aid=i&p_aid=91243448&nummer=702&p_sprache=D&p_indsp=19931512&p_aid=19499442. Reference was last accessed on May 27th 2016.

## Supporting information


**Table S1** Characteristics of the body donors.
