# Supplementary material for: Age‐dependent alterations in osteoblast and osteoclast activity in human cancellous bone
Source: J Cell Mol Med. 2017 Apr 26;21(11):2773–81. doi: 10.1111/jcmm.13192 (PMC5661248; doi:10.1111/jcmm.13192)
Supplement: Supplementary file 1 — Table S1 Characteristics of the body donors. [file JCMM-21-2773-s001.doc]

| **ID** | **age** | **sex** | **cause of death, medical conditions** |
| --- | --- | --- | --- |
| 1 | 90 | F | cardiogenic shock , coronary artery disease (CAD) |
| 2 | 72 | M | multiple organ failure (MOF), prostate cancer (no metastasis), diabetes mellitus, coronary artery disease (CAD) |
| 3 | 81 | M | respiratory insuffiency, pneumonia, gliablastoma (no metastasis) |
| 4 | 62 | M | respiratory failure, pneumonia, esophagus carcinoma (no metastasis) |
| 5 | 75 | F | tumor cachexia, urothelium carcinoma (no metastasis) |
| 6 | 91 | F | pneumonia, dementia |
| 7 | 88 | M | cardiac insuffiency, coronary artery disease (CAD), myocardial infact, renal insuffiency, psychopharmaceutical intake |
| 8 | 71 | F | non-small-cell lung carcinoma (NSCLC) (no metastasis)  Chronic obstructive pulmonary disease (COPD) |
| 9 | 89 | F | myocardial infact, peripheral artery occlusive disease (PAOD),  beta blocker intake |
| 10 | 95 | M | sepsis, terminal renal insuffiency, terminal liver failure, apoplexy, hypertonia |
| 11 | 77 | M | respiratory insuffiency, rectal cancer with brain and lung metastasis, prostatic hyperplasia, arterial hypertension |

**Supplementary table 1: Characteristics of the body donors**
